# Red cell distribution width and Glasgow coma scale score as predictors of in-hospital mortality in maintenance hemodialysis patients diagnosed with spontaneous intracerebral hemorrhage

**DOI:** 10.1097/MD.0000000000031094

**Published:** 2022-10-21

**Authors:** Wen Cao, Haoyuan Ren, Bin Song, Zuchun Liao, Haiyan Li

**Affiliations:** a Department of Nephrology, People’s Hospital of Deyang City, Deyang, Sichuan, China; b Department of Gastrointestinal Surgery, People’s Hospital of Deyang City, Deyang, Sichuan, China.

**Keywords:** GCS score, hemodialysis, prognosis, red blood cell distribution width, spontaneous intracerebral hemorrhage

## Abstract

Glasgow Coma Scale (GCS) score is being widely used as a useful predictor to investigate patients with head injury. High red cell distribution width (RDW) values have been independently associated with mortality and poor neurological outcome. However, there are few data available for Spontaneous Intracerebral Hemorrhage (SIH) in maintenance hemodialysis (MHD) patients. This study aimed to evaluate the prognostic value of the combined measurement of RDW and GCS score in MHD patients with SIH. We retrospectively studied 46 MHD patients who was admitted to our hospital for nontraumatic SIH from October 2014 to May 2020. Data including demographic information, cause of renal failure, comorbidities at ESRD, clinical and laboratory parameters at admission were collected from medical records. Univariate and multivariate Logistic regression analysis were performed to identify independent risk factors of the in-hospital Mortality in Hemodialysis Patients with SIH. The receiver operating characteristic curve (ROC) and areas under the curve (AUCs) were determined. The sensitivity and specificity of independent risk factors were calculated for a range of different cutoff points. A total of 46 patients were enrolled in the study. The in-hospital mortality rate was 69.57%. We divided subjects into 2 groups based on the clinical outcomes. Compared with survivors (n = 14), non-survivors (n = 32) had longer hemodialysis vintage (*P* = .017), lower GCS score (*P* < .001), higher hemoglobin (Hb) (*P* = .032) and RDW (*P* = .009). In multivariate logistic regression analysis, GCS score (OR 0.719, 95% CI 0.546-0.946; *P* = .018) and RDW (OR 4.549, 95% CI 1.243-0.946; *P* = .018) were independent risk factors of in-hospital mortality in MHD patients with SIH. The area under the ROC curve (AUC) for GCS score was 0.849 (95% CI 0.729-0.970) while that for RDW was 0.743 (95% CI 0.596-0.891). The AUC for the combined prediction was 0.916 (95% CI 0.828-1.000), with a sensitivity of 90.63% and a specificity of 88.46%. In conclusion, high RDW and low GCS score were useful and independent poor prognostic markers for in-hospital mortality of MHD patients with SIH.

## 1. Introduction

Nowadays, cerebrovascular events are major causes of morbidity and mortality worldwide.^[1–4]^ spontaneous intracerebral hemorrhage (SIH) is one of the most lethal complications that can occur in maintenance hemodialysis (MHD) patients, which leads to a mortality rate that is approximately 3.8 times higher than that in the general population.^[5]^ Uremia, the pathophysiologic state and the routine use of heparin during hemodialysis can increase the risk of SIH.^[6]^ Moreover, the fluctuations of cerebral hemodynamics such as hypertension or hypotension during the process of dialysis as well as repeated swings of blood pressure occurred frequently in hemodialysis patients can also result in a higher risk for SIH.^[7,8]^ It is well documented that inflammation plays a critical role in brain injury after intracerebral hemorrhage onset.^[9,10]^

Red cell distribution width (RDW) is a marker of variation of the size of the circulating red blood cells, which may reflect an underlying inflammatory state.^[11]^ Previous studies^[12]^ had shown that high RDW values were independently associated with mortality and poor neurological outcome. For ease of application, simplicity and quickness, Glasgow Coma Scale (GCS) score is being widely used as a useful predictor to investigate patients with head injury.^[13–15]^ Previous researches^[16]^ had demonstrated that lower GCS score is an independent risk factor for 30-day mortality in hemodialysis patients with SIH. The RDW and GCS score had been proven to be associated with adverse outcomes in patients with SIH. However, the impact of the combined assessment of the RDW and GCS score levels as a prognostic marker of in-hospital mortality in hemodialysis with SIH is unknown. The aim of the study was to identify the GCS and RDW as predictors of the in-hospital mortality in hemodialysis patients with SIH.

## 2. Methods

### 2.1. Patients and study design

This study was a single-center, retrospective cohort study. We enrolled 46 end-stage renal disease (ESRD) patients treated with MHD for at least 3 months (3 times per week for 4-h sessions), who were admitted to the people’s hospital of Deyang City with a diagnosis of SIH by clinical examinations and cranial computed tomography from October 2014 to May 2020. The patients were followed up for 30 days from the onset of SIH.

Patients with previous neurological disease including trauma, ischemic or hemorrhagic stroke, a history of blood transfusion within 3 months, pregnancy, age <18 years, malignant disease, hematological disease, inflammatory disease were excluded.

### 2.2. Ethics approval and consent to participate

The study protocol was approved by the Ethics Committee of People’s hospital of Deyang City (no. 2021-04-144-K01). Data were collected from electronical patient records. Obtaining informed consent was waived by the Medical Ethics Committee since data were collected and processed anonymously. If patients objected against use of their medical record for research purposes they were not included in the database. We confirm that the study methods were in accordance with the standards formulated by the Declaration of Helsinki and relevant guidelines and regulations.

### 2.3. Clinical assessments

Demographic information, clinical characteristics and laboratory test results were routinely collected on admission including sex, age, hemodialysis vintage, cause of renal failure, comorbidities at ESRD, systemic blood pressure, diastolic blood pressure, mean arterial pressure, GCS score, white blood cell (WBC), neutrophils, lymphocyte, neutrophil to lymphocyte ratio (NLR), hemoglobin (Hb), RDW, platelet (PLT), urea nitrogen (UREA), creatinine (CR), albumin (ALB), calcium (Ca), phosphorus (P), intact parathyroid hormone (iPTH), international normalized ratio, prothrombin time, activated partial thromboplastin time, ferritin, transferrin saturation.

### 2.4. Statistical analysis

SPSS 22.0 statistical software (IBM, Armonk, NY) was used for data analysis. Normally distributed variables including age, systemic blood pressure, diastolic blood pressure, mean arterial pressure, lymphocyte, NLR, Hb, PLT, UREA, CR, ALB, Ca, P, international normalized ratio, prothrombin time, activated partial thromboplastin time, ferritin, transferrin saturation are expressed as mean ± standard deviation. Nonnormally distributed variables including hemodialysis vintage, GCS score, WBC, neutrophils, RDW, iPTH are expressed as median (25%-75% interquartile range). Student *t* test or the Mann–Whitney *U* test was used for comparisons between 2 groups. Categorical variables including sex, cause of renal failure and comorbidities are expressed as number and proportions (percentage), and the Chi-squared test was used for comparisons between 2 groups. Univariate and multivariate Logistic regression analysis were performed to identify independent risk factors of the in-hospital Mortality in Hemodialysis Patients with SIH. The receiver operating characteristic curve (ROC) and areas under the curve (AUCs) were determined. The sensitivity and specificity of independent risk factors were calculated for a range of different cutoff points. *P* values of <.05 was regarded as statistically significant.

## 3. Results

### 3.1. Baseline characteristics

A total of 46 patients were enrolled in the study. There were 33 (71.74%) male patients and 13 (28.26%) female patients, who had a mean age of 60.04 ± 10.70 (range, 39-84) years. 32 patients (69.57%) died during hospitalization. Baseline clinical and laboratory characteristics of the patients were presented in Table 1. Compared with survivors (n = 14), non-survivors (n = 32) had longer hemodialysis vintage (*P* = .017), lower GCS score (*P* < .001), higher Hb (*P* = .032) and RDW (*P* = .009). However, there were no significant differences in sex, cause of renal failure, comorbidities at ESRD, clinical and laboratory parameters including blood pressure, WBC, NLR, PLT, UREA, CR, ALB, Ca, P, iPTH, indices of coagulation and iron metabolism between survivors and non-survivors group (*P* > .05).

**Table 1:** Baseline clinical and laboratory characteristic of subjects at admission.

	Total (n = 46)	Survivors (n = 14)	Non-survivors (n = 32)	*P* value
Male, n	33(71.74)	10(71.43)	23(71.88)	.745
Age, yrs	60.04 ± 10.67	64.00 ± 11.08	58.31 ± 9.86	.090
Hemodialysis vintage, months	31.5(15.8,62.3)	26.0(11.0,49.0)	43.0(20.0,83.0)	.017
**Cause of renal failure**				
Glomerular nephritis	17(36.96)	3(21.43)	14(43.75)	.149
Diabetes	18(39.13)	6(42.86)	13(40.63)	.887
Polycystic kidney disease	7(15.22)	3(21.43)	4(12.5)	.742
Hypertension	3(6.52)	2(14.29)	1(3.13)	.580
**Comorbidities at ESRD**				
Diabetes	19(41.30)	6(42.86)	13(40.63)	.887
Hypertension	43(93.48)	13(92.86)	30(93.75)	.592
Cardiovascular disease	16(34.78)	5(35.71)	11(34.38)	.858
**Clinical and laboratory parameters**				
SBP, mm Hg	187.20 ± 38.04	190.07 ± 29.26	185.94 ± 40.68	.734
DBP, mm Hg	97.89 ± 20.347	98.21 ± 16.58	97.75 ± 21.49	.944
MAP, mm Hg	127.66 ± 24.07	128.83 ± 17.75	127.15 ± 26.00	.826
GCS score	9(5,14.25)	15(13,15)	7(4,13)	.000
WBC, 10^9^/L	7.15(5.05,9.49)	5.35(4.93,7.12)	7.20(5.82,11.79)	.181
Neutrophils, 10^9^/L	5.41(3.95,7.96)	3.97(3.85,5.48)	5.33(4.38,9.89)	.152
Lymphocyte, 10^9^/L	0.87 ± 0.46	0.87 ± 0.54	0.87 ± 0.42	.963
NLR	9.49 ± 6.96	7.34 ± 4.40	10.42 ± 7.70	.170
Hb, g/L	107.04 ± 22.96	96.36 ± 19.93	111.72 ± 22.26	.032
RDW, %	14.6(13.8,15.4)	13.8(13.2,14.6)	15.1(14.4,16.5)	.009
PLT, 10^9^/L	121.00 ± 46.69	127.50 ± 37.79	118.16 ± 49.14	.538
UREA, mmol/L	18.00 ± 6.72	18.17 ± 6.21	17.93 ± 6.82	.924
CR, μmol/L	722.12 ± 224.78	754.92 ± 207.52	707.99 ± 227.09	.511
ALB, g/L	43.09 ± 4.90	41.68 ± 4.61	43.70 ± 4.82	.191
Ca, mmol/L	2.11 ± 0.28	2.09 ± 0.21	2.14 ± 0.29	.592
P, mmol/L	1.35 ± 0.47	1.31 ± 0.35	1.37 ± 0.51	.699
iPTH, pg/ml	323.6(235.5574.5)	352.3(320.8371.5)	364.5(275.6,1094.6)	.496
INR	1.06 ± 0.08	1.03 ± 0.07	1.07 ± 0.08	.230
PT, s	12.09 ± 0.90	11.86 ± 0.80	12.19 ± 0.91	.254
APTT, s	27.24 ± 4.16	26.09 ± 2.82	27.75 ± 4.47	.207
FER, μg/L	318.81 ± 359.61	305.85 ± 186.41	357.60 ± 410.53	.655
TSAT, %	26.95 ± 11.78	24.04 ± 10.90	28.26 ± 11.72	.257

ALB = albumin, APTT = activated partial thromboplastin time, Ca = calcium, CR = creatinine, DBP = diastolic blood pressure, FER = ferritin, GCS score = Glasgow Coma Scale score, Hb = hemoglobin, INR = international normalized ratio, iPTH = intact parathyroid hormone, MAP = mean arterial pressure, NLR = neutrophil to lymphocyte ratio, P = phosphorus, PLT = platelet, PT = prothrombin time, RDW = red cell distribution width, SBP = systemic blood pressure, TSAT = transferrin saturation, UREA = urea nitrogen, WBC = white blood cell.

### 3.2. Risk factors for in-hospital mortality of MHD patients with SIH

Logistic regression was used to analyses the independent risk factors for in-hospital mortality of MHD patients with SIH. Significant factors from the univariate logistic regression analysis were included in the multivariable logistic regression analysis (Table 2). GCS score (OR 0.719, 95% CI 0.546-0.946; *P* = .018) and RDW (OR 4.549, 95% CI 1.243-0.946; *P* = .022) remained statistically significant after adjusting for confounding factors.

**Table 2:** Logistic regression analysis of independent risk factors related to in-hospital mortality of hemodialysis patients with spontaneous cerebral hemorrhage.

	Univariate			Multivariate		
	OR	95%CI	*P* value	OR	95%CI	*P* value
Hemodialysis vintage	1.030	1.001~1.060	.045	1.014	0.975~1.054	.497
GCS score	0.720	0.595~.892	.002	0.719	0.546~0.946	.018
Hb	1.036	1.001~1.072	.044	1.041	0.991~1.095	.110
RDW	2.525	1.218~5.237	.013	4.549	1.243~16.646	.022

GCS score = Glasgow Coma Scale score, Hb = hemoglobin, RDW = red cell distribution width.

### 3.3. The combined predictive value of GCS score and RDW

**Figure 1.**
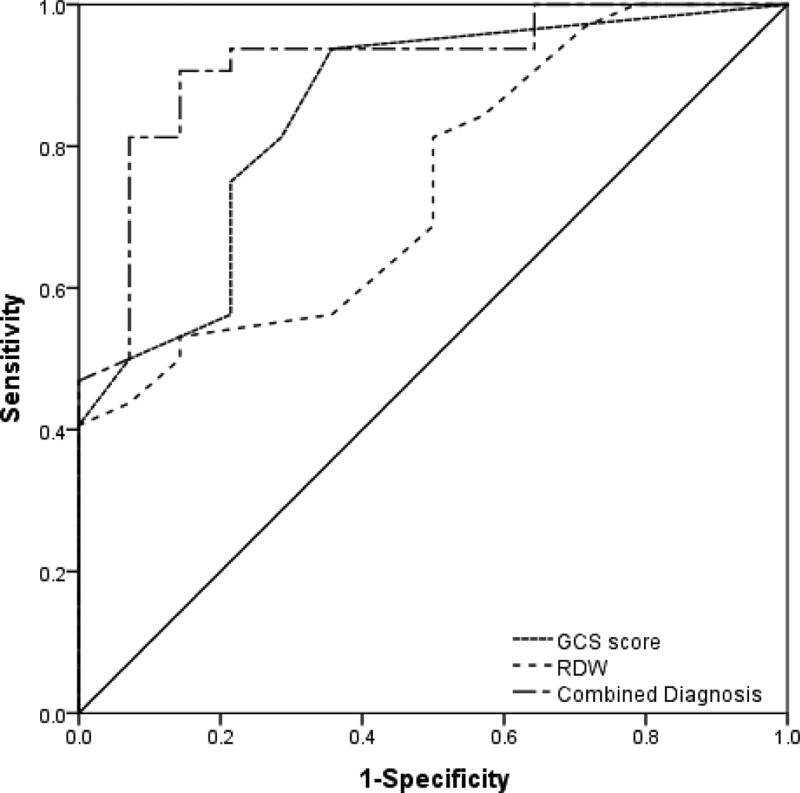
Receiver operator characteristic (ROC) curves for combination of GCS score and RDW. Combination of GCS score and RDW significantly increased AUC in prediction of in-hospital mortality of MHD patients with SIH (all *P* < .05). AUC = areas under the curve, GCS = Glasgow coma scale, MHD = maintenance hemodialysis, RDW = red cell distribution width.

The AUC was used to estimate the predictability of in-hospital mortality of MHD patients with SIH. The AUC for GCS score was 0.849 (95% CI 0.729-0.970) while that for RDW was 0.743 (95% CI 0.596-0.891). In order to get a further understanding whether GCS score and RDW in combination could improve the prognostic performance of MHD patients with SIH, we combined the 2 biomarkers to construct a new ROC curve. The AUC for the combined prediction was 0.916 (95% CI 0.828-1.000), with a sensitivity of 90.63% and a specificity of 88.46% (Table 3 and Fig. 1).

**Table 3:** Diagnostic performance of the GCS score, RDW and the combined marker.

Variables	AUC (95%CI)	Cutoff point	Sensitivity (%)	Specificity (%)	Youden’s index	*P* value
GCS score	0.849(0.729-0.970)	13	81.25	71.43	0.53	<.001
RDW	0.743(0.596-0.891)	14.9	53.13	85.71	0.41	.009
Combined marker	0.916(0.828-1.000)	-	90.63	88.46	0.79	<.001

AUC = areas under the curve, GCS score = Glasgow Coma Scale score, RDW = red cell distribution width.

## 4. Discussion

RDW is a marker of variation of the size of the circulating red blood cells. Higher RDW had been proven to be associated with higher mortality in patients suffering from different diseases such as heart failure,^[17]^ coronary disease,^[18]^ cardiac arrest^[19]^ ischemic stroke,^[20,21]^ sepsis,^[22,23]^ liver disease,^[24]^ or pancreatitis.^[25]^ Previous studies had shown a closely link between RDW and kidney function.^[26–29]^ Also, increased RDW has also been associated with higher mortality in patients with chronic kidney disease (CKD). In 2014, Peng et al^[30]^ demonstrated that RDW ≥ 15.5% was significantly related with cardiovascular disease mortality in peritoneal dialysis patients. A comprehensive meta-analysis from 9 studies which involved 117,047 CKD patients had shown that baseline RDW was a remarkably consistent and strong predictor of all-cause mortality.^[31]^ Another retrospective observational study^[32]^ using a contemporary cohort of 109,675 adult HD patients presented that higher RDW is associated with incrementally higher mortality risk, and it could improve the prognostic performance of the model for 1- and 5-year mortality by nearly 14%. This study also suggested that RDW has a better predictive value for mortality than other traditional anemia laboratory markers in HD patients. RDW is related to not only mortality in patients with CKD, but also poor prognosis in patients with cerebrovascular events. It has been speculated that RDW is a marker of chronic health status but recent studies have shown that elevated RDW may also reflect recent hemorrhage.^[33]^ Elkhatib et al^[34]^ conducted a prospective observational study had shown that the presence of higher RDW was associated with the development of hematoma expansion in the setting of SIH. Similarly, results from a single center with 60 hypertensive intracerebral hemorrhage patients demonstrated a greater admission RDW value was independently associated with hypertensive hematoma growth in the first 12 hours.^[35]^ García et al^[36,37]^ have described the sequential histologic changes that occurred in the hematoma to confirm the relationship between inflammation and intracerebral hemorrhage. The possible mechanisms responsible for the relationship between RDW and SIH including that higher levels of RDW may reflect an underlying inflammatory state.^[11]^ Higher RDW may be a marker of inflammation and may result from the increased immature erythrocytes.^[38]^ Chronic inflammation may promote cell apoptosis, induce myelosuppression, reduce renal production of erythropoietin and bioavailability of iron.^[39,40]^ Hemodialysis patients usually have higher levels of inflammation and oxidative stress because of blood contact with the dialysis membrane, microbial contamination of the dialysate, reduced vitamin C and E levels and reduced activity of the glutathione system,^[32]^ leading to an increase in heterogeneity of RBCs.^[41,42]^ There are some other plausible mechanisms that might explain the RDW related mortality risk. Malnutrition is common in HD patients, which is a independent risk factor associated with RDW elevation.^[43]^ RDW had been shown that RDW is independently related to endothelial dysfunction in patients with CKD.^[44]^

The GCS was the first grading scale to assess the consciousness of patients objectively.^[45]^ Gennarelli et al^[46]^ had demonstrated a relationship between assessments of the GCS Score and the clinical outcomes which showed an association between decreases in GCS Score and increasing mortality. Several studies had shown that lower GCS score is an independent risk factor for unfavorable outcomes in hemodialysis patients with SIH and traumatic intracranial hemorrhage.^[47,48]^

In our study, we found that GCS score and RDW independently predicted in-hospital mortality of hemodialysis patients with SIH. The predictive power of GCS score is stronger than that of RDW. However, GCS also has its limitations as a subjective evaluation indicator. GCS score is difficult to measure patients in sedated, connected to ventilator, with maxillofacial trauma, and injured under the influence of illicit drugs or alcohol.^[49]^ Although the predictive power of RDW was lower as compared with GCS score, the addition of RDW, as an objective indicator, significantly improved the predictive power as demonstrated by AUC (0.916, *P* < .001), and it also slightly improved the sensitivity (90.63%) and specificity (88.46%). Consequently, the combination of RDW and GCS score represented a better biomarker for hemodialysis patients with SAH than RDW and GCS score separately.

There were several limitations of our investigation which should be discussed. Firstly, it was a single-center and retrospective study with a relatively small number of patients so that our population may not reflect the whole cohort. Secondly, the follow-up was limited to the period of hospital stay and the patients were not followed for long-term outcomes after their discharge. Thirdly, since ours was not a randomized trial, the inherent bias was inevitable. It is necessary for further prospective studies with larger patient groups to investigate the combination prognostic value of the 2 parameters.

In conclusion, this study suggested that high RDW and low GCS score were useful and independent poor prognostic markers for in-hospital mortality of hemodialysis patients with SIH.

## Authors’ contributions

**Conceptualization:** Wen Cao, Haoyuan Ren.

**Data collection:** Zuchun Liao, Haiyan Li.

**Data curation:** Zuchun Liao, Haiyan Li.

**Formal analysis:** Wen Cao, Haoyuan Ren.

**Funding acquisition:** Wen Cao, Bin Song.

**Funding support:** Wen Cao, Bin Song.

**Investigation:** Zuchun Liao, Haiyan Li.

**Resources:** Zuchun Liao, Haiyan Li.

**Software:** Bin Song.

**Supervision:** Haoyuan Ren.

**Writing – original draft:** Wen Cao, Zuchun Liao, Haiyan Li.

**Writing – review & editing:** Haoyuan Ren, Bin Song.
